# Uterus transplants and Mexico’s rule of law

**DOI:** 10.1093/jlb/lsae027

**Published:** 2024-12-17

**Authors:** César Palacios-González, Héctor A Mendoza Cárdenas

**Affiliations:** Uehiro Oxford Institute, University of Oxford, Oxford, 16-17 Saint Ebbe's St, Oxford OX1 1PT, UK; Facultad de Trabajo Social y Desarrollo Humano, Universidad Autónoma de Nuevo León, Avenida Universidad SN, Niños Héroes, Ciudad Universitaria, 66450 San Nicolás de los Garza, N.L., Mexico

**Keywords:** fertility treatment, infertility, Mexico, transplantation, uterus, uterus transplants

## Abstract

Uterus transplantation is a new fertility treatment for some women who lack a functioning uterus. The number of countries where these transplants are performed has steadily increased, and in Mexico, there is a growing interest in this procedure among patients, researchers, and clinicians. In this paper we look at Mexico and its legal system in order to determine what is the legal status of uterus transplants, and whether there is a right to them according to Mexican legislation. To achieve this objective, we have organized this paper into four sections. First, we present a brief synopsis of what uterus transplants entail. Second, we carry out a historical overview of uterus transplants in Mexico. Third, we present the federal laws and regulations that apply to uterus transplants in Mexico. Finally, we defend that under Mexican legislation there is a positive right to uterus transplants. We substantiate the former by focusing on the right to the protection of health and the right to family making.

## I. INTRODUCTION

Uterus transplantation (UTx), as a fertility treatment, is a novel procedure that entails a major surgical intervention in combination with assisted reproduction (ie IVF). It was developed to aid women who lack a functional uterus to achieve pregnancy, gestate, and deliver a live child. The first modern UTx happened in 2000 in Saudi Arabia, but unfortunately the uterus had to be removed 99 days after the procedure. The first successful case, meaning UTx plus live delivery was reported in 2014, in Sweden. There are several reasons why a woman’s uterus could not adequately function, some congenital and some iatrogenic. For example, when a woman has had her uterus partially removed due to cancer or abdominal trauma. And where there is impossibility to gestate due to the woman having Mayer–Rokitansky–Küster–Hauser Syndrome, which occurs 1 in 5000 live births. People who suffer from this syndrome commonly have an underdeveloped vagina, and the uterus can be small or entirely absent. In terms of the organ donor, the uterus can come from a living donor or a cadaveric donor.^1^ In contrast with other types of organ donation, once the woman has had one or two children it is highly recommended that the uterus be removed. This is done in order to avoid the ill effects of taking immunosuppressive drugs for an extended period of time.

The pioneer of this technique is Dr Mats Brännström, leader of the Swedish UTx team and now professor of the Department of Obstetrics and Gynaecology at the University of Gothenburg. He and his team have recently published a long-term study on their experience of UTx.^2^ Brännström et al. maintain that UTx can now be described as a safe fertility treatment, for women who lack a functional uterus. The study examined seven successful cases of live uterus donation, out of nine procedures. Of the seven women, six women became pregnant after UTx and in vitro fertilization, resulting in a success rate of 86 per cent. Out of these six women three had two children each, so the total number of births was nine. The Swedish team monitored the growth and development of the babies, during the first 2 years of life, and no adverse effects were found. The researchers plan to monitor the children born from this study at least until adulthood. Brännström and his teams are very optimistic about the results: ‘This is the first comprehensive study to be performed, and the results exceed expectations both in terms of clinical pregnancy rate and cumulative pregnancy rate’.^3^ Currently, there have been more than 70 UTx worldwide, and as of 2021 approximately 50 children have been born after such procedure.^4^ The list of countries where successful UTx has been carried out include: Türkiye, Brazil, USA, Lebanon, India, Spain, China, Czech Republic, Germany, and Sweden.^5^ In Mexico there is a growing interest in this procedure among patients, researchers, and clinicians. In this paper we examine where does UTx fit within the Mexican federal legal system, and whether there is a right to it according to Mexican law and its current implementation. The paper proceeds as follows. In the next section we present a historical survey of UTx in Mexico. In the third section we analyze the legal standing of UTx under Mexican federal law. In the final section we defend that under Mexican federal legislation there is a positive right to UTx, and that said right stems from the right to the protection of health and the right to family making.

## II. UTX IN MEXICO

The Mexican UTx story begins in 2015, when the Director of the Institute of Infertility, Dr Luis Arturo Ruvalcaba Castellón,^6^ organized a symposium on UTx in the city of Guadalajara, in the state of Jalisco. During the 2015 symposium, Dr Mats Brännström presented his work on the topic.^7^ Bears mentioning that in Mexico healthcare centers that have the word ‘Institute’ are most commonly associated with those which are state owned and operated, but this is not the case here. The Mexican Institute of Infertility is a private fertility clinic, which operates in the state of Jalisco.

Between 2015 and 2019, it appears that there were no symposiums or other events related to UTx in Mexico. However, there are at least two academic publications on UTx where Mexican researchers and clinicians, who work in Mexico, appear as co-authors. These are: The History Behind Successful Uterine Transplantation in Humans; and Uterine Autotransplantation in the Nonhuman Primate With Preservation of the Uterine and Ovarian Vascular Pedicles. As its name states, the first paper is a historical account of the first successful UTx in humans.^8^ The second paper evaluates ‘the technical feasibility of performing a uterine autotransplantation in the nonhuman primate while preserving the uterine and ovarian vascular pedicles’.^9^ In addition to this, Mexico, in the context of UTx, is mentioned in two further academic papers. The following appears in a 2016 paper by Jone’s et al.: ‘Now that UTx research teams have been established globally, including teams in France, China, Australia, Singapore and Mexico, an orchestrated approach would certainly optimise progress in this field.’^10^ In a 2018 paper, a Japanese team working on UTx in nonhuman primates mentions: ‘Teams in Singapore, China, Taiwan and Mexico have recently requested our help to set up experiments in nonhuman primate models and we have assisted several of these teams to date.’^11^ It is fair, from the limited published evidence, to conclude that some researchers and clinicians in Mexico are interested in carrying out uterus transplants, are carrying out research on the topic, and that they are in touch with the wider research community.

In February 2019 news broke about the first UTx carried out in Mexico, which occurred on the 18th of said month. According to media reports the surgery lasted 12 hours and the procedure was carried out in a private hospital, Star Medica, in the northern state of Chihuahua. The Mexican news reported that Dr Jaime Arturo Escárcega Preciado and Dr Manuel Gerardo Leal Almeida led the team that carried out the procedure. An interesting fact, which seems not to have been reported in the Mexican news, is that a Spanish clinician was also involved, Dr Francisco Carmona.^12^ The extent of his involvement is unclear; however, the following appeared in Dr Carmona’s personal blog: ‘on the early hours of Tuesday 19^th^ of February (Spanish time) I participated in the first uterus transplant carried out in the city of Chihuahua, in Mexico (…)’.^13^ Carmona’s participation is relevant since he was also involved in the first UTx in Spain, in 2020, which then led to a live birth on the March 10, 2023.^14^

All the clinical information that we have about the Mexican UTx case stems from news articles, TV news segments, news websites, and personal blogs. We know that the organ recipient was a 26-year-old patient with Mayer–Rokitansky–Küster–Hauser syndrome.^15^ In a news segment about the case, aired on the March 22, 2019, Dr Carlos Gastón mentioned a ‘moderate rejection episode’ and stated that they were treating it.^16^ We also know that this was a live donation case, that the organ donor was a 48-year-old woman, and that she had previously given birth to two children. It is unclear if the donor and recipient were related, but at least one media article mentions that they were close. It was also specified that the transplant was logged in the National Registry of Transplants.^17^ Finally, no live births have been reported from this case, and we do not know if the transplanted uterus has been removed or not.

Almost exactly a year after the first transplant, in February 2020, the first protocol for UTx was presented in the state of Jalisco. Even though the protocol was announced by local health authorities it is, unfortunately, not publicly available.^18^ It was presented by the clinicians Juán Luis Contreras Rodríguez^19^ and Luis Arturo Ruvalcaba Castellón, before members of the Technical Committee of the Council of Organ and Tissue Transplantation of Jalisco. Dr Contreras Rodríguez is a co-author in the nonhuman primate UTx paper; and Dr Ruvalcaba Castellón is a co-author both in the nonhuman primate UTx paper, and the history of UTx. Interestingly, Dr Contreras Rodríguez mentioned that in 2019 the Mexican Federal Commission for the Protection Against Sanitary Risks (COFEPRIS) granted permission ‘to carry out a uterus transplant’.^20^ This is an interesting claim since, as we will explain later, the authorization to carry out any type of organ transplant, *qua* medical intervention, falls outside the scope of COFEPRIS’s authority.^21^

Before moving to the next section let us conclude by mentioning that at least two private medical centers in Mexico offer UTx on their websites. The Fertility Centre, in Tijuana, and the Unidad de Trasplantes de Alta Especialidad, in Jalisco.^22^ Furthermore, the Unidad de Trasplantes de Alta Especialidad maintains that it was the first center in Mexico to be certified to carry out such procedure. Its website does not mention who certified them, or when the certification was obtained. It is also of relevance to note that neither center mentions whether UTx is offered as part of ongoing clinical research or as a clinical treatment.

## III. UTX AND THE MEXICAN LEGISLATION

In Mexico, the General Health Law is a federal level piece of legislation that regulates health provision in the country. In terms of hierarchy, it comes after the Political Constitution of the United Mexican States, and the international treaties on the protection of human rights of which Mexico is a signatory.^23^

We will now examine UTx in relation to Chapter III of the General Health Law, which governs organ transplantation.^24^ Article 320 stipulates that every person is the disposer of their body and may donate it, totally or partially, for the purposes and in accordance with the requirements set forth in the third chapter of the General Health Law.^25^ Furthermore, Article 330 permits organ transplants to occur when the results of research carried out for this purpose have been satisfactory, they present an acceptable level of risk to the life and health of the donor and recipient, and provided that there are therapeutic justifications. Based on what we have already said, it is reasonable to conclude that UTx, even when it might still be considered to be part of a research program, fulfills the three criteria required by Article 330.^26^ This article also maintains that the transplant of gonads is forbidden. UTx does not violate this provision, since during the surgical intervention the female gonads, ie the ovaries, are not transplanted.

Article 331 of the General Health Law states that when carrying out organ transplantations, cadaveric donation should be preferred over live donation. This clearly entails that for UTx we should prefer deceased donors, although *live donation is not forbidden*.^27^ However, Article 333 closes the door to certain types of live UTx. It establishes that in order to carry out an organ transplant between live individuals the body of the donor must be able to compensate the functions of the donated organ, in an adequate and safe way.

Article 333.- To carry out transplants between live individuals, the following requirements must be met with respect to the donor:

I. (…)

II. Donate an organ or part of it that, when removed, its function can be compensated by the donor’s body in an adequate and sufficiently safe way; (…).^28^

The rationale of this article is easy to understand when we think about other types of live donations, such as kidney transplants. The law, in such cases, protects the health of the donor by making sure that their other kidney, for example, can make up for the functions of the one removed. This article, thus, closes the door to people donating both kidneys, for example.

If we accept that the biological function of the uterus is to take part in menstruation, embryo implantation, gestation, and labor then we have to conclude that Article 333 forbids uterus donation from *pre-menopausal* women. Because in such instances the donor’s body cannot compensate for the functions of the donated uterus. On the other hand, Article 333 in fact *allows* uterus donation from post-menopausal women, which is a medical possibility in the context of UTx. Let us remember that in the first ever successful case of UTx, the uterus came from a post-menopausal 61-year-old woman donor.^29^ Mexico’s official norm for the prevention and control of diseases in women during perimenopause and postmenopause defines menopause as: ‘a unique event in the life of women, which corresponds to the last menstruation, and is identified after twelve months of amenorrhea’.^30^ If we accept the former definition then when a post-menopausal woman donates her uterus there is no function that needs to be compensated for, since post-menopausal women do not menstruate, there is no embryo implantation, they do not gestate, and do not go into labor. Article 333 can therefore be applied in a straightforward way: live donation of uteri is legal when the donors are post-menopausal, and it is illegal when the donors are pre-menopausal.

That said, we consider that there is at least one good reason for Article 333 to be amended. The reason is that Article 330’s rationale for allowing the donation of uteri by post-menopausal women can also be applied to pre-menopausal women: (i) the results of UTx research have been satisfactory, (ii) there is an acceptable level of risk for the life and health of the donor and recipient, and (iii) there are therapeutic justifications for carrying out the procedure. Since one of the primary objectives of Article 333 is to protect the health of the donor let us elaborate more on this matter. For recipients, UTx complications are most commonly graft failures due to vascular factors.^31^ In a recent clinical review article Veroux et al. list the following as the most common complications for donors: ‘urinary tract complications, infection, bleeding, thrombosis, and hematoma, bowel injuries, urinary tract infections, and iliac vessels and ureter injury’.^32^ However, they note, ‘most of these complications have been prevented with improved skill and expertise in living-donor hysterectomy and with the introduction of mini-invasive techniques, such as robotic hysterectomy’.^33^ One possible way in which Article 333 could be amended is by introducing an exception:

Article 333.- To carry out transplants between live individuals, the following requirements must be met with respect to the donor:

I. (…)

II. Donate an organ or part of it that, when removed, its function can be compensated by the donor’s body in an adequate and sufficiently safe way; *with the exception of the uterus. Women can donate their uteri when they have decided in an informed and free manner that they have fulfilled their childbearing goals*;

There are three further articles of the General Health Law that are important to mention, these are Article 322, Article 323, and Article 327. Article 322 stipulates that when someone decides to donate their organs, they can choose to donate them to specific individuals or institutions.^34^ For example, a mother would be able to directly donate a kidney to her daughter. Article 323 maintains that, an individual’s express consent is required in writing for the live donation of organs and tissues.^35^ And article 327 both prohibits the sale of organs, and establishes that organ donation should be guided by the principles of altruism, non-profit making, and confidentiality.^36^

In addition to federal level legislation, we also need to examine Mexican Official Norms. These norms are mandatory technical regulations issued by the competent agencies (ie competent authorities). In this particular case we need to look into norm NOM-EM- 003-SSA-1994, issued by the Health Secretariat.^37^ This norm regulates the disposal of organs and tissues for therapeutic purposes. According to point 9.2 of the norm, organs in which anastomosis is required and that can be transplanted from a cadaver are: kidney, pancreas, liver, heart, lung, and the small intestine.^38^ Point 9.3 establishes that the organs that can be transplanted from a live donor are: kidney (one), lung (one segment), liver (one segment), pancreas (distal segment), and the small intestine (no more than 50 cm).^39^

Neither point 9.2 nor point 9.3 mention the uterus, therefore even when there are UTx instances that comply with the General Health Law, there is legal uncertainty on whether UTx would run afoul of the official norm. The norm NOM-EM- 003-SSA-1994 could be interpreted as forbidding all but the organ transplants that are explicitly mentioned there. If we were to accept this interpretation then it would follow that UTx is forbidden. However, there is a second possible interpretation of this norm. The second interpretation maintains that since UTx are not explicitly forbidden then it follows that they are not outlawed. One reason for supporting the latter interpretation is that both 9.2 and 9.3 use the following formulation ‘the organs that are susceptible to being transplanted (…)’ and this might lead us to understand the subsequent lists of organs as non-exhaustive ones. There would be no room for ambiguity if 9.2 and 9.3 said ‘the *only* organs that are susceptible to being transplanted (…)’. A further reason to favor the latter interpretation is that, following a 2011 amendment to the federal constitution, Mexico has adopted a broad interpretation approach to the protection of human rights across its legal system.^40^ Consistent with this amendment, as we will argue in Section IV, adopting the latter interpretation of the official norm would provide a broader protection for a woman’s right to the protection of her health. Nevertheless, in order to establish legal clarity regarding UTx in Mexico this norm needs to be amended too. Specifically, uteri would need to be added to the 9.2 and 9.3 lists, by Mexico’s Health Secretariat. The Secretariat can do so since amending such official norm falls within its statutory powers.

Let us revisit the first UTx performed in Mexico, the one which took place in the state of Chihuahua. We can conclude that *there is legal uncertainty* on whether the first UTx in Mexico followed the relevant Mexican Official Norm, specifically point 9.3; and that it is open to further investigation if it also violated the General Health Law, depending on whether the uterus donor was post or pre-menopausal. We also need to ponder how the Chihuahua team were permitted to register this transplant in the National Transplant Registry, since it is unclear whether the procedure is legal. Interestingly, the 2019 UTx does not appear in the Statistics on Donation and Transplantation’s website, of the National Transplant Centre. It does not appear in any of the following: trimestral reports, semestral reports, and annual reports for both 2019 and 2020.^41^ Finally, it is also unclear if the transplant was part of clinical research, or not. However, at this point in time, there is no public evidence that the transplant was indeed part of clinical research. If it had been, then the research would have required express authorization and supervision from the Federal Health Secretariat. According to Article 76 of the Regulations of the General Health Law on Sanitary Control of the Disposal of Organs, Tissues and Corpses of Human Beings:

ARTICLE 76.- Clinical research and teaching in the field of transplants may only be carried out by professionals and in medical institutions that have express authorization and under the supervision of the [Health] Secretariat.^42^

Whether the Chihuahua team received authorization and supervision from the Health Secretariat was not mentioned in any of the media articles on the matter. Furthermore, it is also open to debate whether the Health Secretariat could have authorized the transplant, since it might have violated the relevant official norm: NOM-EM- 003-SSA-1994.

We conclude this section by exploring whether the COFEPRIS could grant permission to carry out a uterus transplant in Mexico. As its name states, COFEPRIS is tasked with protecting Mexicans from sanitary risks and this includes, among many other things, regulating medicines, tobacco, and stem cells. In the previous section we cited Dr Contreras Rodríguez as saying that COFEPRIS had granted them permission to carry out a uterus transplant. However, COFEPRIS does not have the powers to allow an organ transplant *qua* organ transplant. Article 3, subsection 1, of the Regulations of the Federal Commission for the Protection Against Sanitary Risks asserts that COFEPRIS has the power to:

I. Exercise the regulation, control, surveillance and health promotion, which in terms of the applicable provisions correspond to the Secretariat in matters of:

- Establishments: health, for the retrieval of organs, […]^43^

What this article tells us is that part of COFEPRIS’ mandate is to apply the Health Secretariat’s regulations to establishments where organ transplants and retrievals happen. Neither this article, nor any other, states that COFEPRIS can modify or repeal such regulations. Thus, COFEPRIS cannot authorize UTx since they, as part of clinical research, would require express authorization and supervision from the Health Secretariat, according to the previously mentioned Article 76, of the Regulations of the General Health Law on Sanitary Control of the Disposal of Organs, Tissues and Corpses of Human Beings. What might be happening here is that there is confusion between the ability of COFEPRIS to authorize a new type of organ transplant under clinical research, which it does not have, and its ability to put forward the requirements that hospitals that carry out transplants need to meet in order to function. Article 14, subsection 3, of the Regulations of the Federal Commission for the Protection Against Sanitary Risks states that COFEPRIS’s Sanitary Authorization Commission, which is an administrative unit, can:^44^

III. Propose, in coordination with the competent administrative units, the requirements and administrative provisions of a general nature that correspond, for the operation of establishments (…) dedicated to the donation and transplantation of organs, tissues, cells of human beings and their components, and those dedicated to the disposal of blood;

Therefore, the sense in which COFEPRIS could have ‘granted’ permission to carry out a UTx within a clinical research setting would be if it had approved a particular hospital, or clinic, which had met the relevant requirements and administrative provisions for the organ transplantation to take place.

Now, up to this point we have implicitly assumed that UTx in Mexico would fall within clinical research. The reason for this assumption is double. First, UTx is still considered to be a novel intervention that is open to improvement, and this is the case even if we accept that it is a safe fertility treatment.^45^^–^^46^ For example, Brännström et al. maintain that ‘[f]urther studies are needed to develop this novel infertility treatment through optimization of surgical procedures and assisted reproduction treatments, along with enhanced obstetric management and psychological support’.^47^ Second, the General Health Law asserts that health research entails the development of measures that contribute to: (i) the knowledge of biological and psychological processes in humans, (ii) the knowledge of the links between the causes of disease, medical practice, and social structures, (iii) the prevention and control of health problems that are considered a priority for the population, (iv) the knowledge and control of the harmful effects of the environment on health, (v) the study techniques and methods that are recommended or used within the provision of services of health, and (vi) the national production of health supplies.^48^ The development of UTx in Mexico meets at least two of the six conditions. It meets the first condition, in that it increases knowledge of the biological and psychological processes associated with UTx in the Mexican population. And it meets the third condition, in that health problems associated with family planning, such as infertility, are considered a priority for the population. In this regard, the National Centre for Gender Equity and Reproductive Health has as its second priority objective to: ‘Promote the exercise of chosen, protected and healthy sexuality through contraception, family planning, prevention and timely sexual health care’.^49^

## IV. IS THERE A RIGHT TO UTX?

So far we have examined the history of UTx in Mexico, its legal status, and we have provided one good reason for Article 333 to be amended. In this section we turn our attention to the question of whether under Mexican legislation there is a right to UTx. Here we defend that according to Mexican federal law, and its current implementation, there is a positive right to UTx. This further strengthens our case for amending Article 333. We next develop a two-pronged approach to show the existence of such positive right. One prong looks at the right to the protection of health, and the second one looks at the right to form a family. Before moving forward, let us clarify that in this section we do not tackle the *general* philosophical question of whether there is a right to assisted reproductive techniques (ARTs), and if so if this right is a negative or positive one.^50^ Here we limit ourselves to the Mexican legal framework and what follows from it.

### IV.A. Right to the Protection of Health

Article 4 of the Mexican Constitution establishes that:

Article 4.- (…) Every person has a right to the protection of their health. (…)^51^

And Article 1 Bis and Article 2 of the General Health Law assert:

Article 1. Bis.- Health is understood as a state of complete physical, mental and social well-being, and not just the absence of affections or diseases.

Article 2.- The right to health protection has the following purposes:

I. The physical and mental well-being of the person, to contribute to the full exercise of their capacities;

II. The prolongation and improvement of the quality of human life; (…).^52^

In addition to these articles, it is important to note that the Mexico’s Health Secretariat, the World Health Organization, and Christopher Boorse’s biostatistical account of health classify infertility in pre-menopausal women as a disease. Mexico’s Health Secretariat, in its adoption of the CIE-10, maintains that infertility is a disease; and the Mexican Social Security Institute has produced clinical guidelines where infertility is also categorized as a disease.^53^^–^^55^

The WHO maintains that infertility is ‘a disease of the reproductive system defined by the failure to achieve a clinical pregnancy after 12 months or more of regular unprotected intercourse’.^56^ And Boorse’s account of health maintains that:

Health in a member of the reference class [a natural class of organisms of uniform functional design; specifically, an age group of a sex of a species] is *normal functioning*: the readiness of each internal part to perform all its normal functions on typical occasions with at least typical efficiency.^57^

Let us use the former to present an argument for the existence of a right to UTx.

P1: Women have a right to the protection of their health by the Mexican state.

P2: Health protection entails both the physical and mental well-being of the person.

P3: The scope of this protection extends to the complete physical and mental well-being of the individual.

P4: Infertility, in pre-menopausal women, negatively impacts their mental well-being

- There is limited research on mental well-being and infertility in the Mexican context, and none specific to uterine factor infertility. However, a small 2022 study showed that Mexican women with a diagnosis of infertility ‘had moderate symptoms of anxiety and mild symptoms of depression’.^58^ And in a previous qualitative study, emotional distress was an emerging theme in in-depth interviews of infertile women of Mexican descent living in the Tri-state New York.^59^

P5: Infertility, in pre-menopausal women, negatively impacts their physical well-being insofar as it is a disease.

- According to the Mexican State, the WHO, and Boorse’s biostatistical account of health, infertility in pre-menopausal women is a disease.

And from P1 to P5, it follows that

C1: Infertile pre-menopausal women have a right against the Mexican state for it to protect their reproductive health through combating their infertility, this protection encompasses their complete physical and mental well-being.

P6: If infertility negatively impacts women’s mental well-being, when infertility is caused by the lack of a functioning uterus, the lack of a functioning uterus negatively impacts women’s mental well-being.

P7: If infertility impacts women’s physical well-being insofar as it is a disease, when infertility is caused by the lack of a functioning uterus, the lack of a functioning uterus is the cause of a disease.

P8: UTx, at present, is the only way in which the Mexican state could protect the reproductive health, in the sense of acquiring the ability to gestate, of women with uterine factor infertility.

From C1 and P6-P8, we are then entitled to conclude that

C2: Pre-menopausal women with uterine factor infertility have a right to the protection of their health by the Mexican state via UTx.

We now turn our attention to whether such a right is a positive or a negative one under Mexican law. If we were to show that it is a negative right then we would have to accept that the Mexican state has two obligations. First, to amend the relevant laws and official norm so to explicitly include UTx in the lists of approved organ transplants, since this would remove any legal uncertainties (ie amend Article 333 of the General Health Law, and NOM-EM- 003-SSA-1994). Second, the Mexican state has the obligation not to interfere with individuals accessing UTx privately, as long as they follow the relevant laws and regulations. On the other hand, if the right to UTx is a positive one then the Mexican government also has two obligations. The first obligation is, also, to amend the relevant laws and official norm so as to include UTx in the lists of approved organ transplants. The second obligation is to provide the necessary means for women to access UTx. These would include, among other things: training surgeons to conduct UTx, allocating resources for such procedures, and supporting uterus donation.

The Mexican state has adopted, in practice, a positive right stance to reproductive medicine, including ARTs. Since the 1980’s, the public health sector offers, in selected hospitals, ARTs of both low and high complexity at no cost to intended parents and this includes, for example: ovulation induction, and in vitro fertilization.^60^ From this, we can conclude that the Mexican state needs to offer UTx, in order to be consistent with how it treats other areas of reproductive medicine. Now, the pace at which the state should offer this procedure and the resources that it needs to allocate to it are separate issues from whether the Mexican state has an obligation to offer UTx.

### IV.B. Right to Form a Family

Article 4 of the Mexican Constitution states that the law will protect the organization and development of the family, and that individuals have the right to choose the timing and number of children that they want to have.

Article 4.- Man and woman are equal under the law. The law shall protect the organization and development of the family.

Every person has the right to decide in a free, responsible, and informed manner about the number and spacing of their children.^61^

This article clearly states that every woman has a right to choose the number and spacing of their children. We can conclude that there is a right to UTx, from this article, in the following way.

P1. Mexican law shall protect the development of the family.

P2. The development of the family entails the right to decide ‘in a free, responsible, and informed manner about the number and spacing of their children’.

And from P1 and P2, it follows that

C1. The Mexican state shall protect the right of pre-menopausal women with uterine factor infertility to decide ‘in a free, responsible, and informed manner about the number and spacing of their children’.

P3. In order for pre-menopausal women with uterine factor infertility to exercise their right to decide ‘in a free, responsible, and informed manner about the number and spacing of their children’ they would need access to UTx.

From C1 and P3, we are then entitled to conclude that

C2. The Mexican state shall provide access to UTx for pre-menopausal women with uterine factor infertility so they can exercise the right to decide ‘in a free, responsible, and informed manner about the number and spacing of their children’.

We do not have to spend any time exploring whether ‘provide access’ here entails a negative, or positive, right to UTx. We do not need to do so because the Mexican state is required by law to adopt a positive right stance to UTx. In order to see this, let us pay attention to Chapter VI of the General Health Law. This particular chapter is entitled: Services of Family Planification. Article 67 of the General Health Law maintains:

Article 67.- Family planning is of a priority nature. (…) The services provided in this area [family planification] constitute a means for the exercise of the right of every person to decide in a free, responsible, and informed manner about the number and spacing of their children, with full respect for their dignity.^62^

And Article 68 states:

Article 68.- Family planning services include:

(…)

The care and vigilance of acceptors and users of family planning services;

(…)

IV. The support and promotion of research in contraception, human infertility, family planning and biology of human reproduction; ^63^

Research into human infertility entails research on UTx, since such transplants can alleviate some types of uterine factor infertility, as explained in the previous sections. Let us thus stop here for a moment and pay attention to the fact that according to Article 68 the Mexican state is in fact required to support and promote medical research on UTx. Subsection V of Article 68 also tells us that:

V. Participation in the establishment of suitable mechanisms for the determination, preparation, acquisition, storage and distribution of medicines and other supplies for family planning services.^64^

Articles 67 and 68 maintain that the Mexican state, through the Health Secretariat, has an obligation to provide the care and required elements for people to exercise the right to have, or not, children. Thus, the Mexican state is required to adopt a positive right stance to the right to choose the number and spacing of children. Now, given that UTx can be part of the family planning process, as previously explained, and that they are akin to other high complexity interventions that the state already provides (eg in vitro fertilization), then we have to conclude that the positive right to decide the number and spacing of children also entails a positive right to UTx. As said above, the fact that there is such right is different from questions regarding resource allocation and the pace of implementation.^65^

## V. CONCLUSION

In this paper we have shown that the Mexican state is required by law to support and promote medical research on UTx, and that clinical research on UTx requires explicit authorization and supervision from the federal Health Secretariat.^66^ We have also shown that, in the context of live donation, the General Health Law maintains that UTx is legal if the donor is a post-menopausal woman, and illegal if the donor is a pre-menopausal woman. However, the overall legality of UTx is uncertain, since the appropriate Mexican Official Norm does not mention uteri in the lists of approved organs for transplantation, both for live and cadaveric donations. We also concluded that COFEPRIS cannot authorize uterus transplants, *qua* novel medical interventions. Finally, we defended that women in Mexico have a positive right to UTx, stemming from the right to the protection of health and the right to family making. In order for the Mexican state to fulfill its obligations it would need to modify the Mexican Official Norm NOM-EM- 003-SSA-1994 and include uteri in the lists of approved organs for transplantation; and it would need to modify Article 333 of the General Health Law to include a special provision for UTx in the context of live donation.

## Footnotes

1 Emily H. Frisch et al., *Uterus Transplantation: Revisiting the Question of Deceased Donors versus Living Donors for Organ Procurement*, 11 J. Clin. Med. 4516 (2022). Veroux, Massimiliano, et al. *Living-Donor Uterus Transplantation: A Clinical Review*, 13 J. Clin. Med. 775 (2024), https://doi.org/10.3390/jcm13030775

2 Mats Brännström et al., *Reproductive, Obstetric, and Long-Term Health Outcome after Uterus Transplantation: Results of the First Clinical Trial*, 118 Fertil. Steril. 576 (2022).

3
*Id.*

4 For a detailed account of the procedure and its history see: Mats Brännström et al., *Uterus Transplantation: From Research, through Human Trials and into the Future*, Hum. Reprod. Update dmad012 (2023).

5 Randa Akouri et al., *First Live Birth after Uterus Transplantation in the Middle East*, 25 Middle East Fertil. Soc. J. 1 (2020); Mats Brännström et al., *Livebirth after Uterus Transplantation*, 385 The Lancet 607 (2015); Mats Brännström, Michael A. Belfort & Jean Marc Ayoubi, *Uterus Transplantation Worldwide: Clinical Activities and Outcomes*, 26 Curr. Opin. Organ Transplant. 616 (2021); Sara Yvonne Brucker et al., *Living-Donor Uterus Transplantation: Pre-, Intra-, and Postoperative Parameters Relevant to Surgical Success, Pregnancy, and Obstetrics with Live Births*, 9 J. Clin. Med. 2485 (2020); Dani Ejzenberg et al., *Livebirth after Uterus Transplantation from a Deceased Donor in a Recipient with Uterine Infertility*, 392 The Lancet 2697, 9 (2018); Jiri Fronek et al., *Live Birth Following Uterine Transplantation From a Nulliparous Deceased Donor*, 105 Transplantation (2021), https://journals.lww.com/transplantjournal/fulltext/2021/05000/live_birth_following_uterine_transplantation_from.23.aspx; Yanhong Huang et al., *Report of the First Live Birth after Uterus Transplantation in People’s Republic of China*, 114 Fertil. Steril. 1108 (2020); Jessica Mouzo, *Nace en España el primer bebé tras un trasplante de útero*, El Pais, May 22, 2023, https://elpais.com/salud-y-bienestar/2023-05-22/nace-el-primer-bebe-tras-un-trasplante-de-utero-en-espana.html (accessed Aug. 28, 2023); Omer Ozkan et al., *Birth of a Healthy Baby 9 Years after a Surgically Successful Deceased Donor Uterus Transplant*, 275 Ann. Surg. 825 (2022); Pankaj P. Khelka, *Woman Delivers Baby after Getting Womb Transplant from Mother in Pune Hospital*, India Today, Oct. 19, 2018, https://www.indiatoday.in/india/maharashtra/story/woman-delivers-baby-after-getting-womb-transplant-from-mother-in-pune-hospital-1370346-2018-10-18; Shailesh Puntambekar et al., *Novel Anastomotic Technique for Uterine Transplant Using Utero-Ovarian Veins for Venous Drainage and Internal Iliac Arteries for Perfusion in Two Laparoscopically Harvested Uteri*, 26 J. Minim. Invasive Gynecol. 628 (2019); G. Testa et al., *First Live Birth after Uterus Transplantation in the United States*, 18 Am. J. Transplant. 1270 (2018).

6 Dr Ruvalcaba Castellón was also the first physician to carry out, in Mexico, an autologous ovary transplant, back in 2013.

7 Montserrat Muñoz, *Trasplante de Útero: Los Primeros Pasos En México*, CienciaMX Noticias, Mar. 29, 2016, http://www.cienciamx.com/index.php/ciencia/salud/6177-trasplante-de-utero-los-primeros-pasos-en-mexico.

8 Luis Arturo Ruvalcaba Castellón et al., *The History behind Successful Uterine Transplantation in Humans*, 21 Jbra Assist. Reprod. 126 (2017).

9 Michelle N. Han et al., *Uterine Autotransplantation in the Nonhuman Primate with Preservation of the Uterine and Ovarian Vascular Pedicles*, 26 Reprod. Sci. 1329 (2019)

10 B. P. Jones et al., *Uterine Transplantation: Past, Present and Future*, 123 BJOG Int. J. Obstet. Gynaecol. 1434 (2016).

11 Iori Kisu et al., *Basic Research on Uterus Transplantation in Nonhuman Primates in Japan*, 44 J. Obstet. Gynaecol. Res. 1871 (2018).

12 Equipo del Dr Francisco Carmona, *Primer Trasplante de Útero En México*, (2019), https://www.drfcarmona.com/primer-trasplante-de-utero-en-mexico/.

13 La madrugada del martes 19 de febrero (hora española) participé en el primer trasplante de útero realizado en la ciudad de Chihuahua en México…

14 Clínic Barcelona, *Nace El Bebé de La Primera Mujer Trasplantada de Útero En España*, (2023), https://www.clinicbarcelona.org/noticias/nace-el-bebe-de-la-primera-mujer-trasplantada-de-utero-en-espana.

15 Samara Martínez, *Realizan En Chihuahua El Primer Trasplante de Útero En El País*, El Sol de México, Mar. 5, 2019, https://www.elsoldemexico.com.mx/republica/sociedad/realizan-en-chihuahua-el-primer-trasplante-de-utero-en-el-pais-3144041.html.

16 Azteca Uno, *Trasplante de Útero y La Oportunidad de Ser Madre En México*, (2019), https://www.facebook.com/AztecaNoticias/videos/trasplante-de-útero-y-la-oportunidad-de-ser-madre-en-méxico/402329890584685/.

17 Ximena Mejía, *Realizan Primer Trasplante de Útero En México*, Imagen Radio, Mar. 7, 2019, https://www.imagenradio.com.mx/realizan-primer-trasplante-de-utero-en-mexico.

18 Comunicación Social, *Presentan Protocolo Para Abrir Trasplante de Útero en Jalisco*, (2020), https://ssj.jalisco.gob.mx/prensa/noticia/8852.

19 Some news articles mistakenly call him José Luis Contreras Rodríguez.

20 Anonymous, *Jalisco, primer estado de México con protocolo para trasplante de útero*, El Hospital*,* Feb. 10, 2020), https://www.elhospital.com/es/noticias/jalisco-primer-estado-de-mexico-con-protocolo-para-trasplante-de-utero

21 Comunicación Social, *supra* note 18.

22 The Fertility Center, *Transplante de Útero* (2021), https://www.thefertilitycentermexico.com/trasplante-de-utero/ (accessed May 24, 2023); Unidad de Trasplantes de Alta Especialidad, *Trasplante de Útero, Guía Médica*, (2017), http://unitae.mx/trasplante-de-utero/.

23 In this paper we will only focus on UTx and Mexican federal laws, regulations, and norms. Thus, we leave other avenues for showing that there is a right to UTx unexplored. For example, we will not explore the implications for UTx that stem from the ruling of the Inter-American Court of Human Rights on the case Artavia Murillo y otros (‘Fecundación in Vitro’) contra Costa Rica.Inter-American Court of Human Rights, Case of Artavia Murillo et al. (‘In Vitro Fertilization’) v. Costa Rica (2012), http://www.corteidh.or.cr/docs/casos/articulos/seriec_257_ing.pdf (accessed June 12, 2016).

24 Cámara de Diputados del H. Congreso de la Unión, *Ley General de Salud* (1984), http://www.diputados.gob.mx/LeyesBiblio/pdf/142_010616.pdf.

25 Ley General de Salud (*supra* note 24), Artículo 320.- Toda persona es disponente de su cuerpo y podrá donarlo, total o parcialmente, para los fines y con los requisitos previstos en el presente Título.

26 Ley General de Salud (*supra* note 24), Artículo 330.- Los trasplantes de órganos, tejidos y células en seres humanos vivos podrán llevarse a cabo cuando hayan sido satisfactorios los resultados de las investigaciones realizadas al efecto, representen un riesgo aceptable para la salud y la vida del donante y del receptor, y siempre que existan justificantes de orden terapéutico. Está prohibido: I. El trasplante de gónadas o tejidos gonadales

27 Ley General de Salud (*supra* note 24), Artículo 331. La procuración y extracción de órganos o tejidos para trasplantes se hará preferentemente de sujetos en los que se haya comprobado la pérdida de la vida.

28 Ley General de Salud (*supra* note 24), Artículo 333.- Para realizar trasplantes entre vivos, deberán cumplirse los siguientes requisitos respecto del donante: I. (…) II. Donar un órgano o parte de él que al ser extraído su función pueda ser compensada por el organismo del donante de forma adecuada y suficientemente segura;

29 Bill Chappell, *A First: Uterus Transplant Gives Parents A Healthy Baby*, NPR (2014), https://www.npr.org/sections/thetwo-way/2014/10/04/353691555/a-first-uterus-transplant-gives-parents-a-healthy-baby (accessed Aug. 24, 2023).

30 Secretaría de Salud, *NORMA Oficial Mexicana NOM-035-SSA2-2012, Para La Prevención y Control de Enfermedades En La Perimenopausia y Postmenopausia de La Mujer. Criterios Para Brindar Atención Médica*, Diario Oficial de la Federación, Jan. 7, 2013, https://dof.gob.mx/nota_detalle.php?codigo=5284235&fecha=07/01/2013&print=true (accessed May 29, 2023).

31 Iori Kisu et al., *Graft Failure after Uterus Transplantation in 16 Recipients: A Review*, 12 J. Clin. Med. (2023).

32 Veroux, Massimiliano, et al. *Living-Donor Uterus Transplantation: A Clinical Review*, 13 J. Clin. Med. 775 (2024). https://doi.org/10.3390/jcm13030775

33
*Id.*

34 Ley General de Salud (*supra* note 24), Artículo 322.- […] En la donación expresa podrá señalarse que ésta se hace a favor de determinadas personas o instituciones. También podrá expresar el donante las circunstancias de modo, lugar y tiempo y cualquier otra que condicione la donación.

35 Ley General de Salud (*supra* note 24), Artículo 323.- Se requerirá que el consentimiento expreso conste por escrito: I. Para la donación de órganos y tejidos en vida, y II. Para la donación de sangre, components sanguíneos y células troncales en vida.

36 Ley General de Salud (*supra* note 24), Artículo 327. Está prohibido el comercio de órganos, tejidos y células. La donación de éstos se regirá por principios de altruismo, ausencia de ánimo de lucro y confidencialidad, por lo que su obtención y utilización serán estrictamente a título gratuito.

37 Secretaría de Salud, *Norma Oficial Mexicana de Emergencia NOM-EM- 003-SSA-1994, Para La Disposición de Órganos y Tejidos de Seres Humanos Con Fines Terapéuticos, Excepto Sangre y Sus Componentes*, Diario Oficial de la Federación, Sep. 30, 1994, https://www.dof.gob.mx/nota_detalle.php?codigo=4747620&fecha=30/09/1994#gsc.tab=0.

38 NOM-EM- 003-SSA-1994 (*supra* note 44), 9.2 Los órganos susceptibles de ser trasplantados que requieren anastomosis vascular, que se pueden obtener de cadáveres son los siguientes: 9.2.1 Riñón; 9.2.2 Páncreas; 9.2.3 Hígado; 9.2.4 Corazón; 9.2.5 Pulmón, y 9.2.6 Intestino delgado.

39 NOM-EM- 003-SSA-1994 (*supra* note 44), 9.3 Los órganos susceptibles de ser trasplantados que requieren de anastomosis vascular que se pueden obtener de disponentes originarios vivos son los siguientes: 9.3.1 Riñón, uno; 9.3.2 Pulmón, un lóbulo; 9.3.3 Hígado, un lóbulo; 9.3.4 Páncreas, segmento distal, y 9.3.5 Intestino delgado, un segmento no mayor de 50 cm.

40 Maria de Jesús Medina-Arellano, *The Regulation of Human Germline Genome Modification in Mexico*, *in* Human Germline Modification and the Right to Science: A Comparative Study of National Laws and Policies 129 (Andrea Boggio, Cesare P. R. Romano & Jessica Almqvist eds., 1st ed. 2020), https://www.cambridge.org/core/books/abs/human-germline-genome-modification-and-the-right-to-science/regulation-of-human-germline-genome-modification-in-mexico/C3F607541D14DAA0457BDA5C206456F6.

41 CENATRA, Estadísticas- Estadísticas sobre donación y trasplantes, https://www.gob.mx/cenatra/documentos/estadisticas-50060, (accessed Oct. 17, 2024).

42 Cámara de Diputados del H. Congreso de la Unión, *Reglamento de La Ley General de Salud En Materia de Control Sanitario de La Disposición De Organos, Tejidos y Cadáveres de Seres Humanos* (1985), https://www.salud.gob.mx/unidades/cdi/nom/compi/rlgsmcsdotcsh.html. (accessed Oct. 17, 2024). Articulo 76.- La investigación y docencia clínica en materia de trasplante sólo podrá realizarse por profesionales y en instituciones médicas que cuenten con autorización expresa y bajo la vigilancia de la Secretaría.

43 Vicente Fox Quesada, *Reglamento de La Comisión Federal Para La Protección Contra Riesgos Sanitarios*, Diario Oficial de la Federación, Apr. 13, 2004, http://www.ordenjuridico.gob.mx/Documentos/Federal/pdf/wo88332.pdf (accessed May 27, 2023). ARTÍCULO 3. Para el cumplimiento de su objeto, la Comisión Federal tiene a su cargo las siguientes atribuciones: I. Ejercer la regulación, control, vigilancia y fomento sanitarios, que en términos de las disposiciones aplicables corresponden a la Secretaría en materia de: a. Establecimientos: de salud, de disposición de órganos (…)

44
*Id.* III. Proponer, en coordinación con las unidades administrativas competentes, los requisitos y las disposiciones administrativas de carácter general que correspondan, para la operación de establecimientos (…) dedicados a la donación y el trasplante de órganos, tejidos, células de seres humanos y sus componentes, y los dedicados a la disposición de sangre;

45 In fact, UTx is only offered outside of a clinical trial in two centres: Baylor University Medical Center, and University of Alabama at Birmingham Medicine. UAB Medicine, *Uterus Transplant Program,*  https://www.uabmedicine.org/medical-professionals/clinical-services/transplant/uterus-transplant-program/ (accessed Oct. 17, 2024). Baylor Scott & White, *Health Uterus Transplant,*  https://www.bswhealth.com/treatments-and-procedures/uterus-transplant (accessed Oct. 17, 2024). We thank a reviewer for bringing this to our attention.

46 There is a further issue that we will not address here, this is: How does the Mexican legislation approaches medical interventions that in other countries are approved, and have long passed clinical trials, but are new to the country?

47 Brännström et al., *supra* note 2 at 583.

48 Ley General de Salud (*supra* note 24) Artículo 96. La investigación para la salud comprende el desarrollo de acciones que contribuyan: I. Al conocimiento de los procesos biológicos y psicológicos en los seres humanos; II. Al conocimiento de los vínculos entre las causas de enfermedad, la práctica médica y la estructura social; III. A la prevención y control de los problemas de salud que se consideren prioritarios para la población; IV. Al conocimiento y control de los efectos nocivos del ambiente en la salud; V. Al estudio de las técnicas y métodos que se recomienden o empleen para la prestación de servicios de salud, y VI. A la producción nacional de insumos para la salud.

49 Centro Nacional de Equidad de Género y Salud Reproductiva, *Objetivos Prioritarios, https://www.gob.mx/salud/cnegsr/documentos/objetivos-prioritarios* (accessed Oct. 17, 2024)

50 For an in depth discussion of this issue see Giulia Cavaliere & César Palacios-González, *Lesbian Motherhood and Mitochondrial Replacement Techniques: Reproductive Freedom and Genetic Kinship*, J. Med. Ethics (2018).

51 Congreso Constituyente, *Constitución Política de Los Estados Unidos Mexicanos*, (1917), http://www.diputados.gob.mx/LeyesBiblio/htm/1.htm. Artículo 4o. (…) Toda Persona tiene derecho a la protección de la salud.

52 Ley General de Salud (*supra* note 24), Artículo 1o. Bis.- Se entiende por salud como un estado de completo bienestar físico, mental y social, y no solamente la ausencia de afecciones o enfermedades. Artículo 2o.- El derecho a la protección de la salud, tiene las siguientes finalidades: I. El bienestar físico y mental de la persona, para contribuir al ejercicio pleno de sus capacidades; II. La prolongación y mejoramiento de la calidad de la vida humana; (…)

53 Secretaria de Salud, *Catálogo Diagnósticos*, http://www.dgis.salud.gob.mx/contenidos/intercambio/diagnostico_gobmx.html (accessed Oct. 18, 2024)

54 Instituto Mexicano Del Seguro Social, Diagnóstico De La Pareja Infértil Y Tratamiento Con Técnicas De Baja Complejidad (2012), http://www.imss.gob.mx/sites/all/statics/guiasclinicas/621GRR.pdf (accessed Oct. 18, 2024).

55 For a previous discussion regarding infertility in the Mexican context see: César Palacios-González & María de Jesús Medina-Arellano, *Mitochondrial Replacement Techniques and Mexico’s Rule of Law: On the Legality of the First Maternal Spindle Transfer Case*, 4 J. L. Biosci. 50–69 (2017), and César Palacios-González & María de Jesús Medina-Arellano, *Author’s Response to Peer Commentaries: Mexico’s Rule of Law and MRTs*, J. L. Biosci. 623–629 (2017).

56 Fernando Zegers-Hochschild et al., *The International Committee for Monitoring Assisted Reproductive Technology (ICMART) and the World Health Organization (WHO) Revised Glossary on ART Terminology, 2009*, 24 Hum. Reprod. 2683 (2009).

57 Christopher Boorse, *Health as a Theoretical Concept*, 44 Philos. Sci. 542, 555 (1977).

58 Sara Minory Méndez González et al., *Impacto psico-social en mujeres mexicanas que viven con infertilidad*, 25-2 Revista Electrónica de Psicología Iztacala 706, 706 (2022).

59 Janet Brito, *Infertility among Women of Mexican Descent Living in the United States: A Phenomenological Analysis on the Emotional and Sexual Impact and Coping Styles* (2014), https://www.proquest.com/docview/1648740325/previewPDF/9A025A759C6749F3PQ/1?accountid=13042&sourcetype=Dissertations%20&%20Theses

60 Rosa Verónica Esparza-Pérez, *La Depreciación de Los Derechos Humanos y La Regulación de La Reproducción Humana Asistida En México*, 153 Gac. Médica México 626 (2017).

61 Constitución Política de Los Estados Unidos Mexicanos (supra note 58), Artículo 4o. La mujer y el hombre son iguales ante la ley. Ésta protegerá la organización y el desarrollo de la familia. Toda persona tiene derecho a decidir de manera libre, responsable e informada sobre el número y el espaciamiento de sus hijos.

62 Ley General de Salud (*supra* note 24), Artículo 67.- (…) Los servicios que se presten en la materia [de planificaión famiilar] constituyen un medio para el ejercicio del derecho de toda persona a decidir de manera libre, responsible e informada sobre el número y espaciamiento de los hijos, con pleno respeto a su dignidad.

63 Ley General de Salud (*supra* note 24), Artículo 68.- Los servicios de planificación familiar comprenden: (…) II. La atención y vigilancia de los aceptantes y usuarios de servicios de planificación familiar; (…) IV. El apoyo y fomento de la investigación en materia de anticoncepción, infertilidad humana, planificación familiar y biología de la reproducción humana;

64 Ley General de Salud (*supra* note 24), V. La participación en el establecimiento de mecanismos idóneos para la determinación, elaboración, adquisición, almacenamiento y distribución de medicamentos y otros insumos destinados a los servicios de planificación familiar.

65 In this paper we have shown that there is a positive right to UTx. However, this right, as other rights, is not absolute. It does not, for example, entitle infertile women to other women’s uteri. And, in most instances and due to resource scarcity, this positive right will enter into conflict with other individuals’ positive rights to healthcare. The conflict might be, for example, between providing someone with a lifesaving treatment or someone else with UTx. How, then, should the Mexican State act when there is such conflict, or when there is resource scarcity? In a recent writ of amparo (Amparo en Revisión 378/2014), on a matter regarding the right to the protection of health of patients with HIV/AIDS, the Second Chamber of the Supreme Court of Justice of the Nation stated: ‘The ICESCR [International Covenant on Economic, Social and Cultural Rights] itself recognizes that the State’s obligation to protect, respect and promote the human right to the enjoyment of the highest attainable standard of health cannot ignore the particular situation of each country, so that there will be no violation of economic, social and cultural rights, even if it is proven that a particular right has not been fully realized or has not reached an optimal state of effectiveness, as long as the State has demonstrated that it has used all the resources at its disposal in an effort to satisfy the conventional obligations, since in no way is it intended that each country immediately comply with the full realization of the right to health, but rather that, in accordance with the principle of progressiveness, it should take appropriate measures to ensure, as expeditiously and effectively as possible, this objective’. Alberto Pérez Dayán, *Amparo en Revisión*
*378/2014*, Suprema Corte de Justicia de la Nación, Segunda Sala, sentence 15 Oct. 2014, México. Furthermore, authors like Esparza Pérez and Cano Valle argue for a similar position when discussing access to ARTs: ‘This [access] does not entail that public resources are used in an excessive manner to address this issue, but rather to seek that this service is available in a progressive and gradual manner, in addition to the fact that the admission criteria for its programs do not constitute requirements that violate human rights.’ Rosa Verónica Esparza Pérez & Cano Valle Fernando*, The Legal Anchoring to the Techniques of Assisted Reproduction,* L-151 Boletín Mexicano de Derecho Comparado 44, 13–50 (Jan.-Apr. 2018). Even when we cannot provide a full treatment of this matter here, we find the application of the principle of progressiveness in situations of resource scarcity to be a pragmatic and sensible practical solution when introducing life-enhancing interventions. We thank an anonymous reviewer for raising this important issue.

66 Some readers might be wondering if the arguments so far explored extend to trans women. Addressing this issue requires its own dedicated article, and here, due to space considerations, we cannot make it justice. However, we can confidently say that UTx for trans women would not meet, at present, the requirements of the Regulations of the General Health Law on Transplantation, specifically Article 17. Because, at present, it is unknown if UTx would be an effective intervention for trans women. Article 17 states: ‘A person will be considered a candidate to receive a Transplant when: I. He or she has a condition that can be treated effectively by means of an Organ, Tissue or Cell Transplant; (…)’. Cámara de Diputados del H. Congreso de la Unión, *Reglamento de La Ley General de Salud en Materia De Trasplantes* (2014). We thank an anonymous reviewer for raising this important issue.

